# The first complete chloroplast genome sequence of the medicinal plant *Bletilla formosana* (Orchidaceae)

**DOI:** 10.1080/23802359.2019.1700841

**Published:** 2019-12-13

**Authors:** Bo-nian Liao

**Affiliations:** Department of Chinese medicine, Affiliated Hospital of Chuanbei Medical College, Nanchong, China

**Keywords:** *Bletilla formosana*, chloroplast, Illumina sequencing, phylogeny

## Abstract

*Bletilla formosana* is a medicinal plant commonly used in southwest of China. In this study, we sequenced the complete chloroplast (cp) genome sequence of *B. formosana* to investigate its phylogenetic relationship in the family Orchidaceae. The chloroplast genome of *B. formosana* was 159,112 bp in length with 37.3% overall GC content, including a large single-copy (LSC) region of 86,838 bp, a small single-copy (SSC) region of 18,672 bp and a pair of inverted repeats (IRs) of 26,801 bp. The cp genome contained 116 genes, including 83 protein-coding genes, 29 tRNA genes, and 4 rRNA genes. The phylogenetic analysis indicated the genus *Bletilla* was closely related to *Platanthera*.

*Bletilla* is a small genus of the Orchidaceae family, which includes six species in the world. Most of them are mainly distributed in mainland China and Japan (Chen et al. 2009). The roots of plants in this genus, namely ‘seven white’ in Chinese, have been widely used in traditional Chinese medicine for thousands of years (Jiangsu New Medical College 1977). In Chinese pharmacopeia (2015), the species *Bletilla striata*, was the only original species of the ‘seven white’ and recorded in that. But in southwest China, the roots of the species *B. formosana* are commonly used as the ‘seven white’ in local medicine for the treatment of bleeding, colds, esophagitis, erosive gastritis, and burns (Guan et al. 2005). However, up to now many studies have mainly focused on describing its chemical compositions (Guan et al. 2005), pharmacological studies (Zhao et al. 2013) and DNA barcoding analysis (Wu et al. 2014; Song et al. 2017) for such medicinal plant, with little involvement in its genomic analysis, so that no comprehensive genomic resource is conducted for it. Here, we report the chloroplast genome sequence of *B. formosana* and find its internal relationships within the family Orchidaceae.

Fresh and clean leaf materials of *B. formosana* were collected from Wenchuan county, Sichuan, China (N31°4′42.49″, E103°30′38.73″), and the plant materials and a voucher specimen (No. LBN08) were stored in the Department of Chinese medicine, Affiliated Hospital of Chuanbei Medical College. Total genomic DNA was extracted using the improved CTAB method (Doyle 1987; Yang et al. 2014), and sequenced with Illumina Hiseq 2500 (Novogene, Tianjin, China) platform with pair-end (2 × 300 bp) library. The raw data was filtered using Trimmomatic v.0.32 with default settings (Bolger et al. 2014). Then, paired-end reads of clean data were assembled into circular contigs using SOAPdenovo2 (Luo et al. 2012) with *Bletilla striata* (No. NC_028422) as reference. Finally, the cpDNA was annotated by the Dual Organellar Genome Annotator (DOGMA; http://dogma.ccbb.utexas.edu/) (Wyman et al. 2004) and tRNAscan-SE (Lowe and Chan 2016) with manual adjustment using Geneious v. 10.0.3 (Kearse et al. 2012).

The circular genome map was generated with OGDRAW v.1.3.1 (Greiner et al. 2019). Then, the annotated chloroplast genome was submitted to the GenBank under the accession number MN562087. The total length of the chloroplast genome was 159,112 bp, with 37.3% overall GC content. With typical quadripartite structure, a pair of IRs (inverted repeats) of 26,801 bp was separated by a small single-copy (SSC) region of 18,672 bp and a large single-copy (LSC) region of 86,838 bp. The cp genome contained 116 genes, including 86 protein-coding genes, 29 tRNA genes, and 4 rRNA genes. Of these, 20 genes (*rps19*, *trnH-GUG*, *rpl2*, *rpl23*, *trnI-CAU*, *ycf2*, *ycf15*, *trnL-CAA*, *ndhB*, *rps7*, *trnV-GAC*, *rrn16*, *trnI-GAU*, *ycf68*, *trnA-UGC*, *rrn23*, *rrn4.5*, *rrn5*, *trnR-ACG*, *trnN-GUU*) were duplicated in the inverted repeat regions, 8 genes (*rps16*, *atpF*, *rpoC1*, *clpP*, *petB*, *petD*, *rpl16*, *rpl2*), and 6 tRNA genes (*trnK-UUU*, *trnG-GCC*, *trnL-UAA*, *trnV-UAC*, *trnI-GAU*, trnA-UGC) contain one intron, while two genes (*ycf3* and *rps12*) have two introns.

To investigate its taxonomic status, a total of 26 cp genome sequences of Orchidaceae species were downloaded from the NCBI database used for phylogenetic analysis. After using MAFFT V.7.149 for aligning (Katoh and Standley 2013), a neighbor-joining (NJ) tree was constructed in MEGA v.7.0.26 (Kumar et al. 2016) with 1000 bootstrap replicates and two Burmanniaceae species (*Burmannia coelestis*: KT734618, *Burmannia disticha*: KT734619) were used as outgroups. The results showed that the genus *Bletilla* was closely related to *Platanthera* (Figure 1). Meanwhile, the phylogenetic relationship in Orchidaceae was consistent with previous studies and this will be useful data for developing markers for further studies.

**Figure 1.**
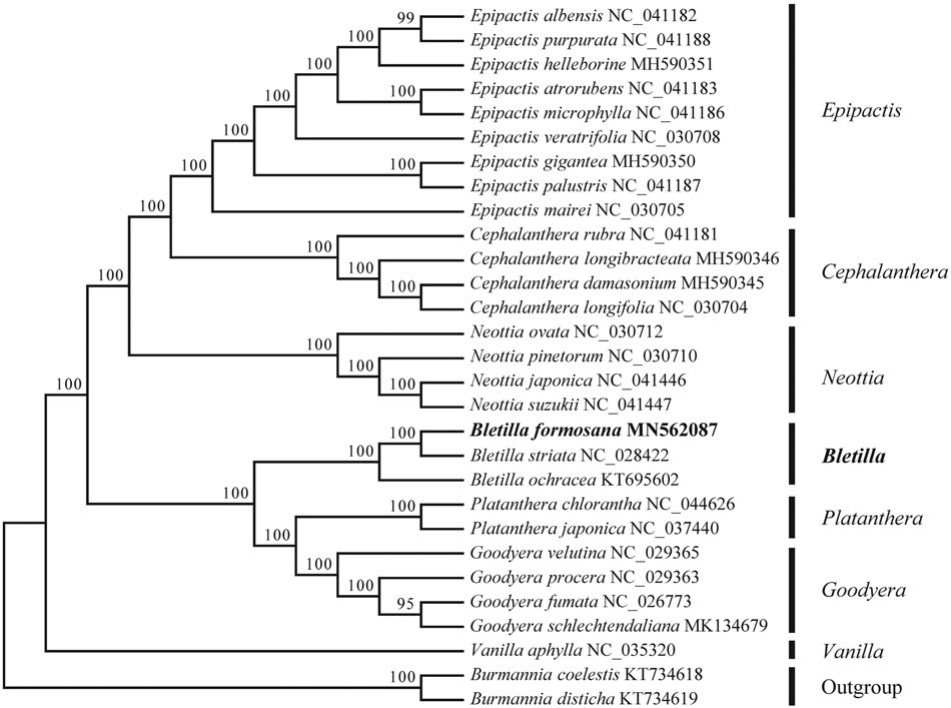
Neighbor-joining (NJ) tree of 27 species within the family Orchidaceae based on the plastomes using two Burmanniaceae species as outgroups.
